# Supplementary material for: An approach to characterize mechanisms of action of anti-amyloidogenic compounds in vitro and in situ
Source: NPJ Parkinsons Dis. 2025 May 10;11:122. doi: 10.1038/s41531-025-00966-5 (PMC12065871; doi:10.1038/s41531-025-00966-5)
Supplement: Supplementary file 1 — Supplementary Figures [file 41531_2025_966_MOESM1_ESM.docx]

**Supplementary Figures**

**An approach to characterize mechanisms of action of anti-amyloidogenic compounds *in vitro* and *in situ***

**
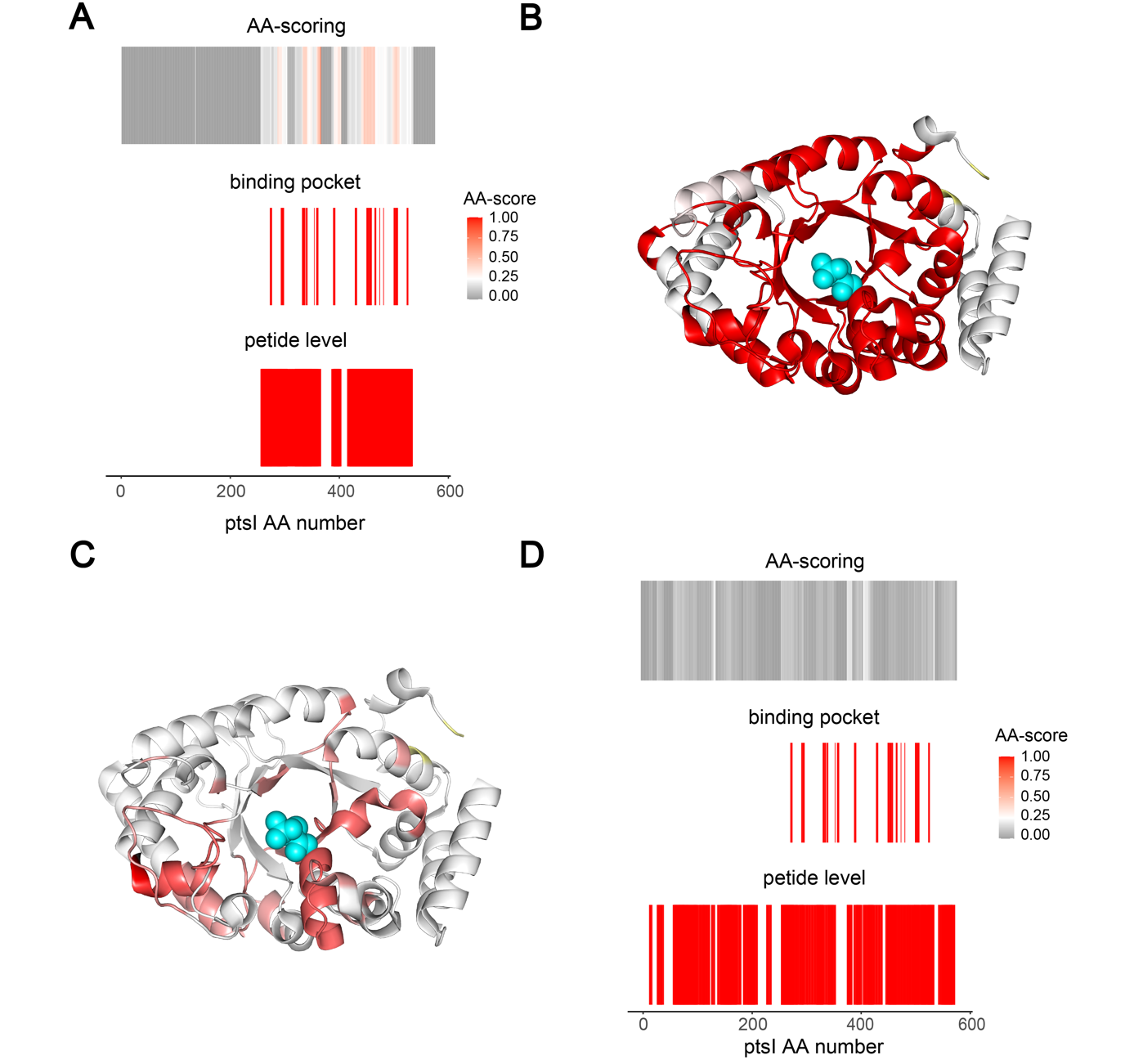
**

**Supplementary figure 1. *In silico* LiP-MS of FBP-ptsI binding.** To assess whether the achieved resolution of the amino acid centric analysis in FBP-ptsI binding was dependent on the specific peptides identified in this experiment, we performed an *in silico* LiP-MS experiment. We computed all potentially detectable peptides of ptsI upon proteinase K and trypsin digestion, with a minimal length of six amino acids and maximally one missed trypsin cleavage. Next, we defined the FBP binding pocket to include all atoms in a sphere of 10 Å radius around PEP. If a computed LiP peptide mapped to the binding pocket, we assigned it a score of 1, otherwise a score of 0. Peptides with a score of 1 are expected to change in a LiP-MS experiment. In an in silico amino acid-level analysis, we then averaged the assigned values per amino acid position. To test the sensitivity of the peptide-level and amino acid-level approaches to false positives, we assigned 5 % of the computed LiP peptides, picked randomly, as significantly changing (score of 1). **A** In silico amino acid centric fingerprint (top), binding pocket (middle) and peptide centric fingerprint (bottom) aligned along the ptsI sequence. **B** *in silico* peptide centric fingerprint mapped on the ptsI structure (PDB: 2xz7). **C** *in silico* amino acid centric fingerprint mapped on the ptsI structure (PDB: 2xz7). **D** Control *in silico* amino acid centric fingerprint (top)and peptide centric fingerprint (bottom) aligned along the ptsI sequence, using random peptides (5% of total peptides) assigned as significantly changing. Binding pocket defined as in A.


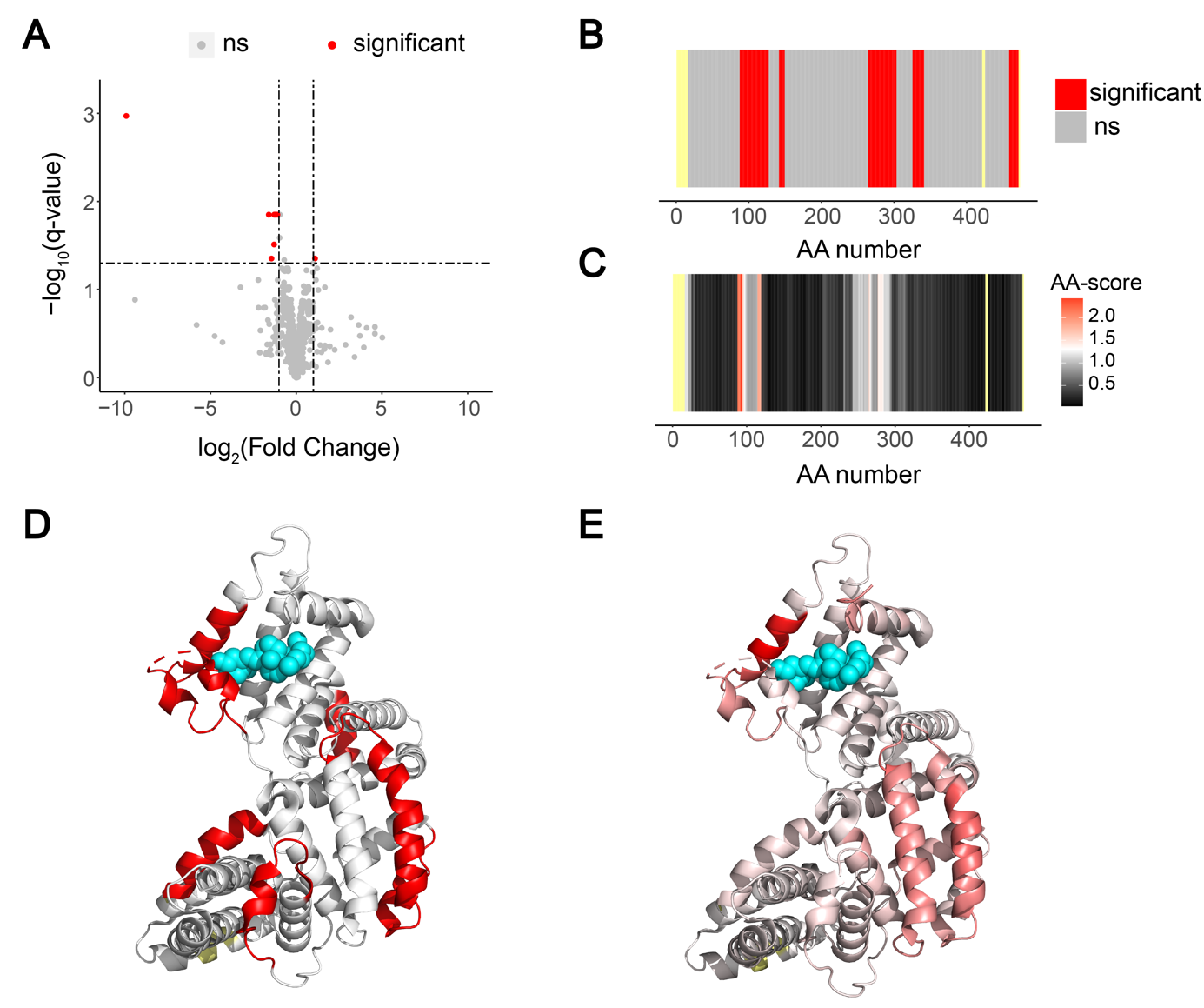


**Supplementary figure 2. Vitamin D binding towards Vitamin D binding protein (GC).** **A** volcano plot comparing peptide abundances of peptides generated in Vitamin D bound and unbound GC. **B** fingerprint of the classical LiP-MS data analysis pipeline. Significant regions in red, not significant in grey, not detected in yellow. **C** fingerprint upon scoring changes per amino acid. The scale indicates the score per amino acid. The significance threshold of -log_10_(0.05) x log_2_(2) is shown in white, with red indicating higher scores. The more intense the red colour, the higher the score. Not significant in grey. Not detected in yellow. **D** significant peptides mapped on the vitamin D binding protein structure (PDB: 1j78). Calcifediol in cyan. **E** significant amino acids of amino acid centric analysis mapped on the vitamin D binding protein structure (PDB: 1j78). Calcifediol in cyan.


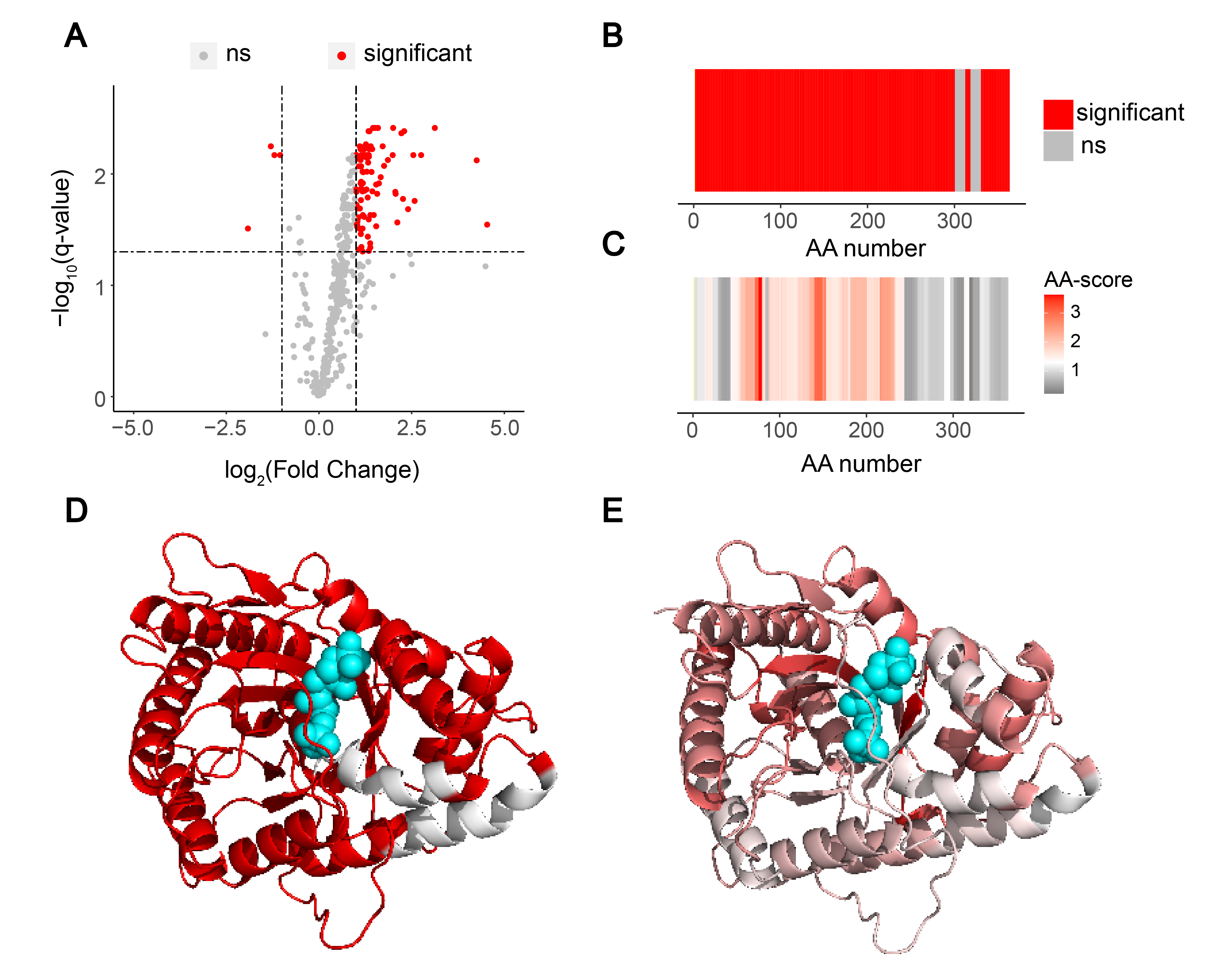


**Supplementary figure 3. Fructose bisphosphate binding towards Fructose-bisphosphate aldolase A (ALDOA).** **A** volcano plot comparing peptide abundances of peptides generated in fructose bisphosphate bound and unbound ALDOA. **B** fingerprint of the classical LiP-MS data analysis pipeline. Significant regions in red, not significant in grey, not detected in yellow. **C** fingerprint upon scoring changes per amino acid. The scale indicates the score per amino acid. The significance threshold of -log_10_(0.05) x log_2_(2) is shown in white, with red indicating higher scores. The more intense the red colour, the higher the score. Not significant in grey. Not detected in yellow. **D** significant peptides mapped on the ALDOA protein structure (PDB: 4ald). Fructose bisphosphate in cyan. **E** significant amino acids of amino acid centric analysis mapped on the ALDOA protein structure (PDB: 4ald). Fructose bisphosphate in cyan.


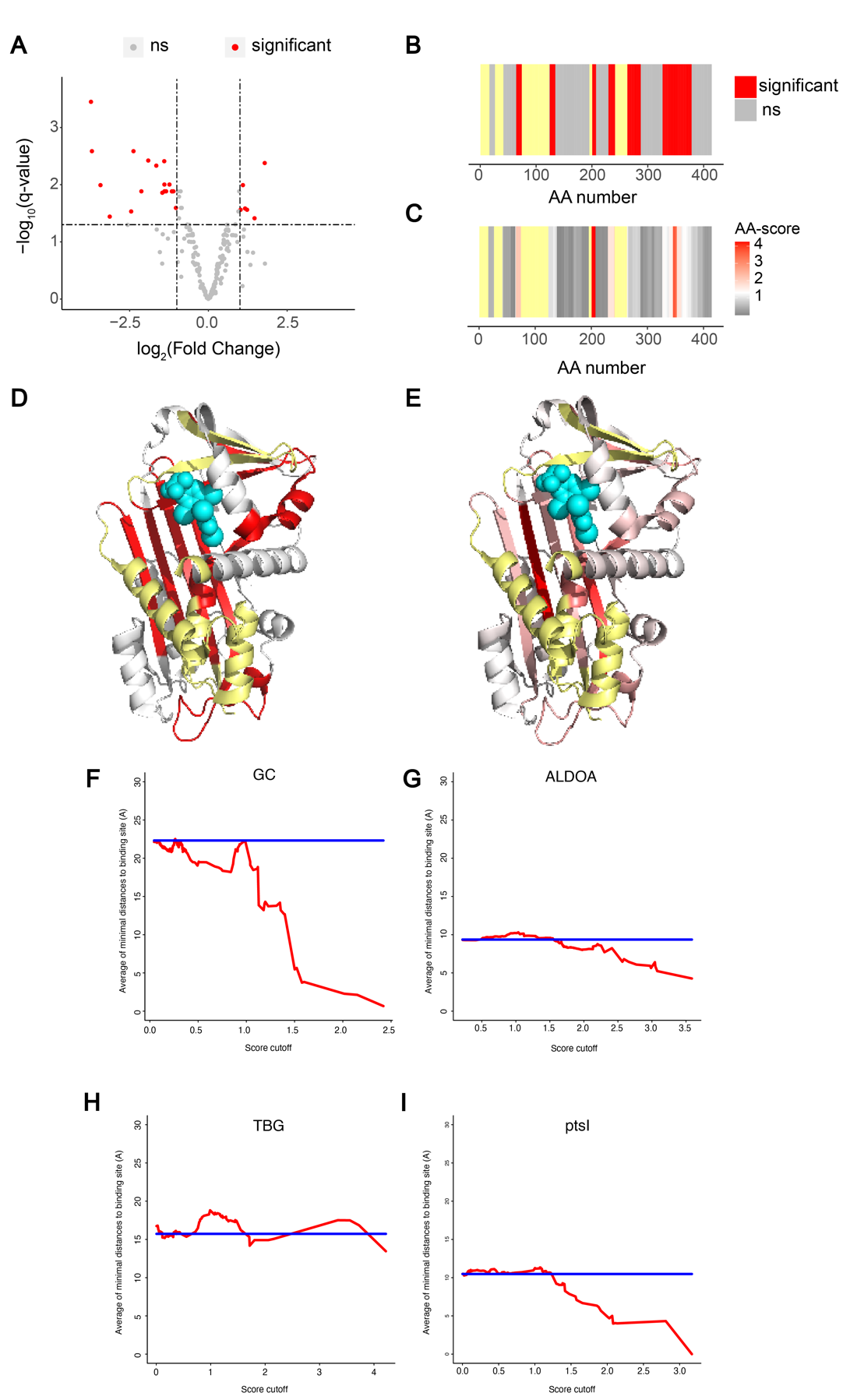


**Supplementary figure 4. Thyroxine binding towards Thyroxine Binding Globulin (TBG). A** volcano plot comparing peptide abundances of peptides generated in thyroxine bound and unbound TBG. **B** fingerprint of the classical LiP-MS data analysis pipeline. Significant regions in red, not significant in grey, not detected in yellow. **C** fingerprint upon scoring changes per amino acid. The scale indicates the score per amino acid. The significance threshold of -log_10_(0.05) x log_2_(2) is shown in white, with red indicating higher scores. The more intense the red colour, the higher the score. Not significant in grey. Not detected in yellow. **D** significant peptides mapped on the TBG protein structure (PDB: 2riw). Thyroxine in cyan. **E** significant amino acids of amino acid centric analysis mapped on the TPG protein structure (PDB: 2riw). Thyroxine in cyan. (**F-I**) The plots show the average minimal distance to the ligand binding site of amino acids with scores above the indicated threshold values, for GC (**F**), ALDOA (**G**), TBG (**H**), and ptsl **(I**) in red. The blue line shows the average minimal distance to the binding site for all amino acids in a protein.


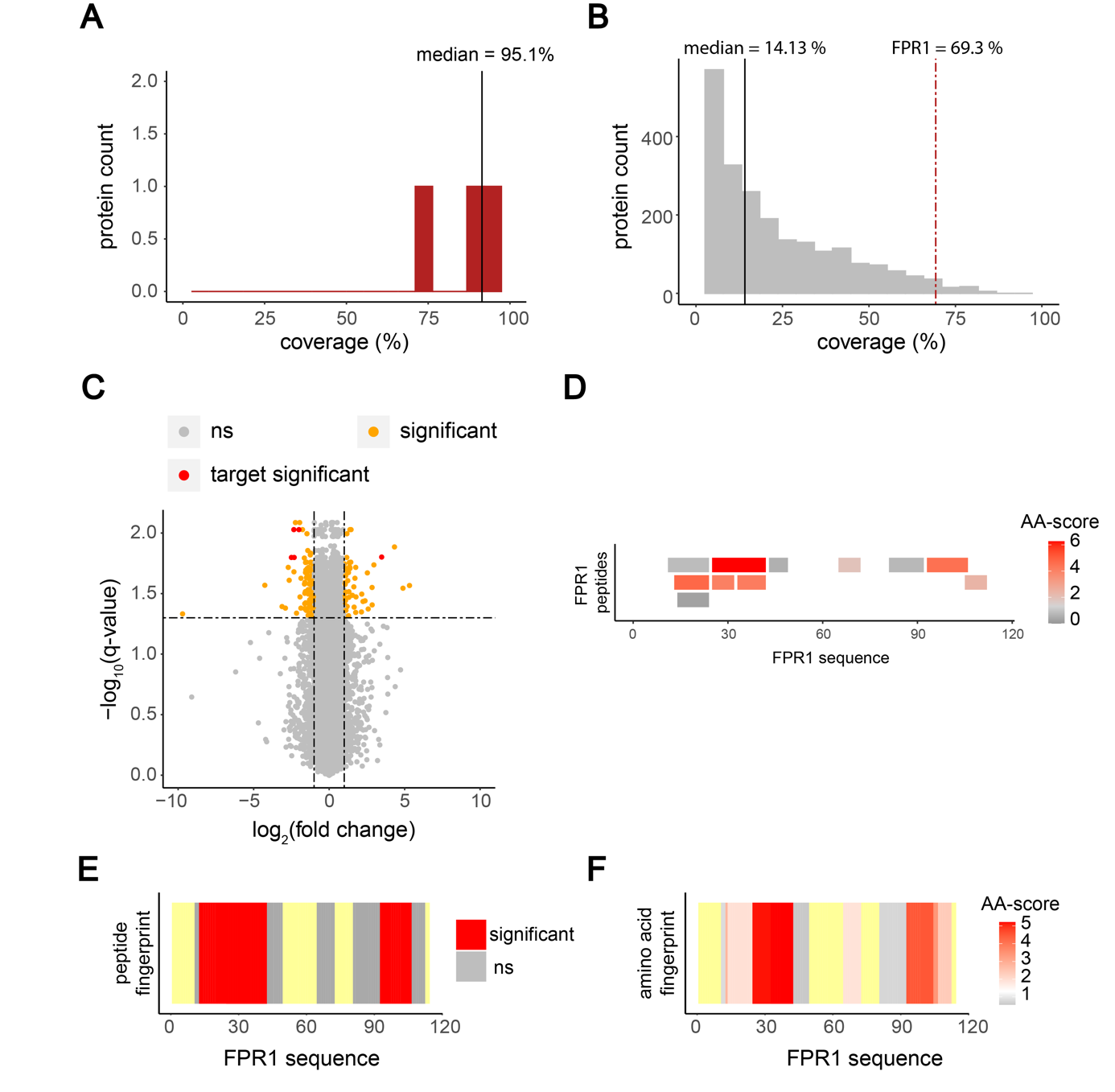


Supplementary figure 5. Amino acid centric analysis on a proteome wide scale. A Coverage of purified proteins analysed with *in vitro* LiP-MS (bin size of 5%). Median coverage is indicated by the black line (95.1%). B Coverage distribution of the 2417 identified proteins (bin size of 5%). Median coverage indicated by the black line (14.13 %). Coverage of FPR1 (69.3%) indicated by the red dotted line. C Volcano plot of rapamycin treated and untreated *S. cerevisiae* cell extracts (non-significant in light grey, significant in orange, significant and FPR1 in red). D Peptides and their corresponding scores (-log_10_(q-value) x absolute (log_2_(fold change)) mapped along the FPR1 sequence. Score values from grey to red. E Peptide-centric analysis showing significant peptides mapped along the sequence of FPR1 and onto the FK-506 bound FPR1 structure (PDB: 1yat). FK-506 in cyan. F Fingerprint upon amino acid centric analysis mapped along the sequence of FK-506 bound FPR1 (The scale indicates the score per amino acid. The significance threshold of -log_10_(0.05) x log_2_(2) is shown in white, with red indicating higher scores. The more intense the red colour, the higher the score. Not significant in grey.) and onto the FK-506 bound FPR1 structure (PDB: 1yat). FK-506 in cyan.


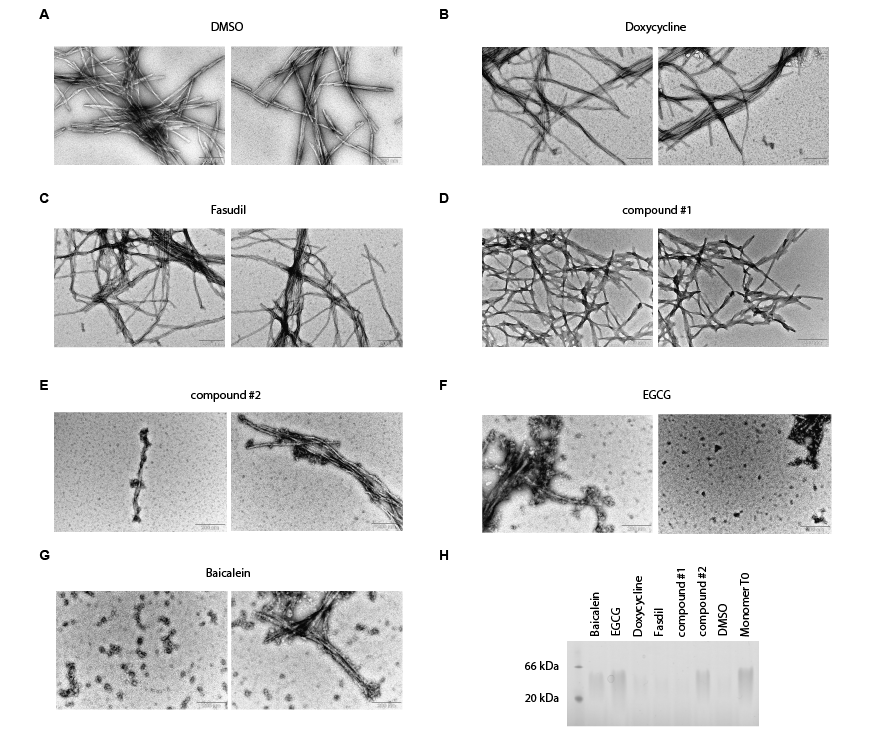


Supplementary figure 6. Transmission electron microscopy images of structures formed in the presence of compounds or DMSO after 17 hours of incubation. A DMSO control condition. B Doxycycline. C Fasudil. D compound 1. E compound 2. F EGCG. G Baicalein. H Native PAGE after 17 hours of incubation in the indicated conditions, compared to monomer at time point 0. Oligomeric and fibrillar forms are not resolved.


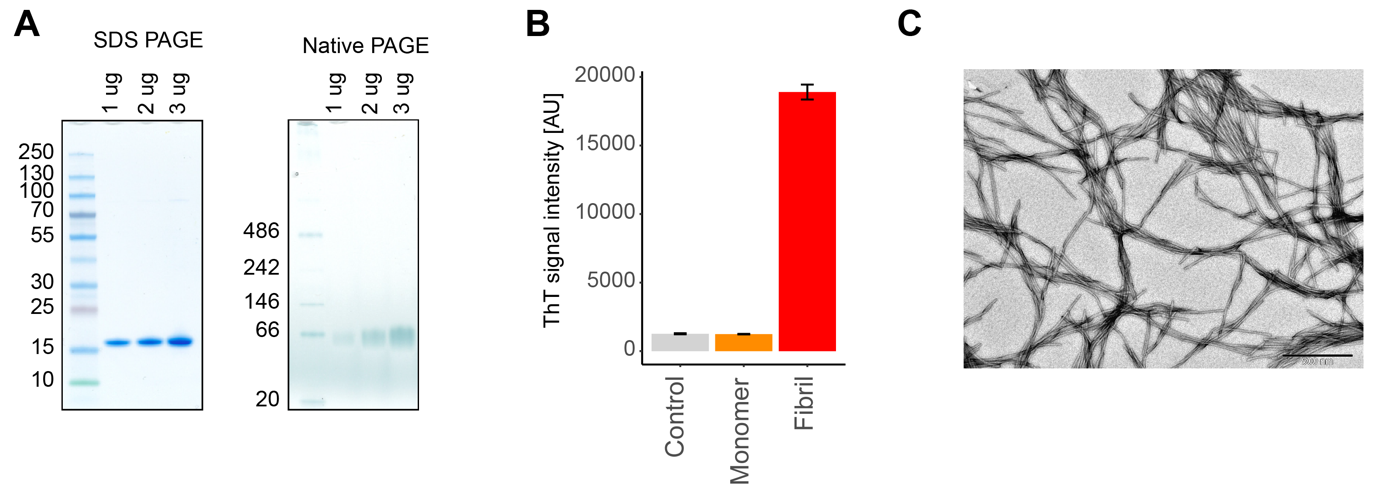


**Supplementary figure 7. Quality controls α-Synuclein monomer and fibrils. A** SDS PAGE and Native PAGE of monomeric α-Synuclein. **B** ThT intensity of Control, monomeric-and fibrillary α-Synuclein. **C** TEM image of the α-Synuclein fibrils used.

**
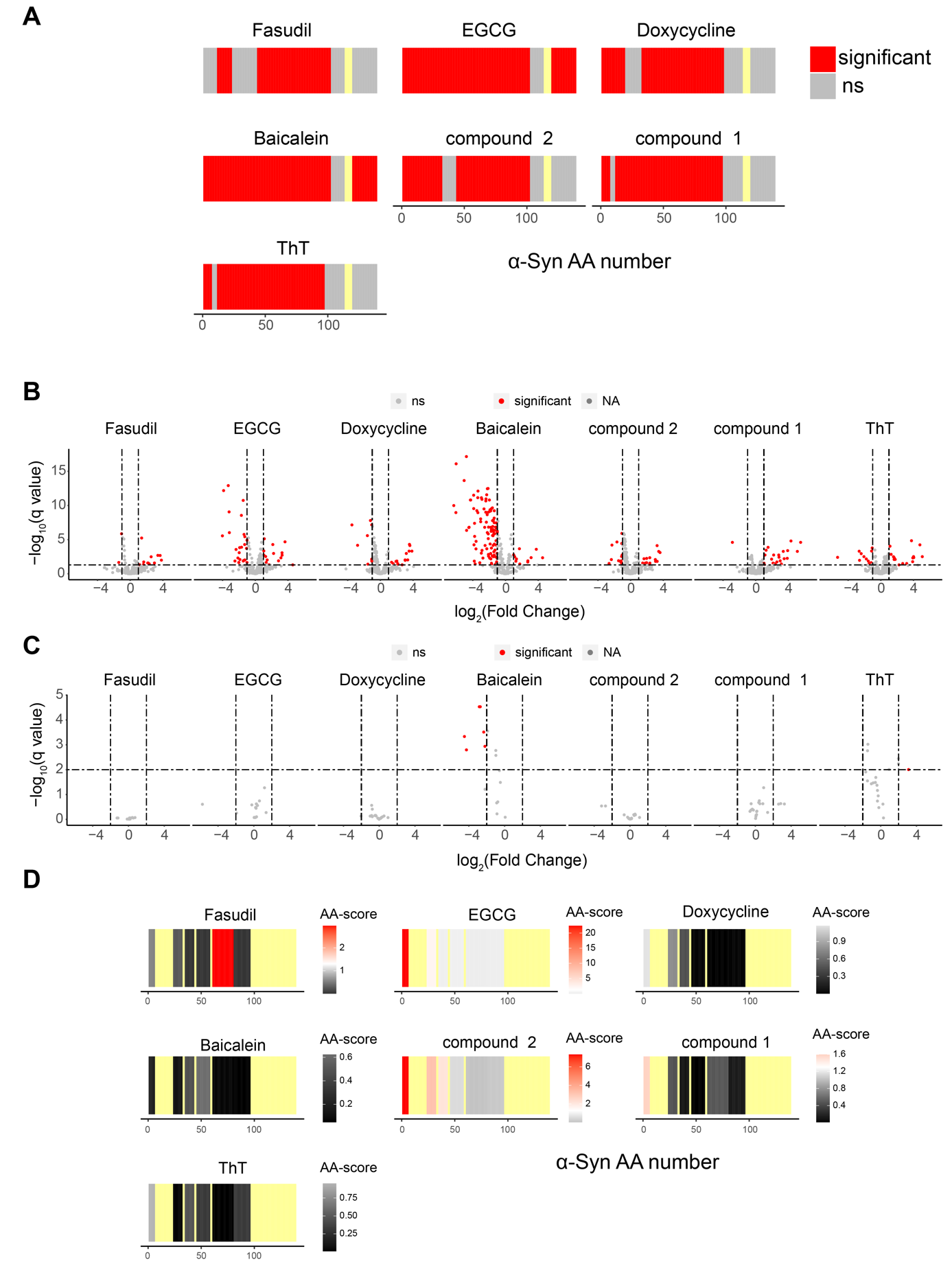
**

**Supplementary figure 8. Compound interactions with α-Synuclein monomer. A, B** LiP peptide fingerprints (non-significant in light grey, significant in red, not detected in yellow) (**A**) and volcano plots of LiP peptide intensities (significant in red, non-significant in grey) **(B**) for α-Synuclein monomer treated with compounds compared to treatment with the DMSO control. **C** Volcano plot comparing the control (i.e., trypsin-only) peptide intensities of compound-treated α-Synuclein monomer to DMSO-treated monomer; colours as in B. **D** LiP peptide fingerprints of α-Synuclein treated with compounds and normalized for the trypsin-only control data (The scale indicates the score per peptide. The significance threshold of -log_10_(0.05) x log_2_(2) is shown in white, with red indicating higher scores. The more intense the red colour, the higher the score. Not significant in grey. Not detected in yellow).


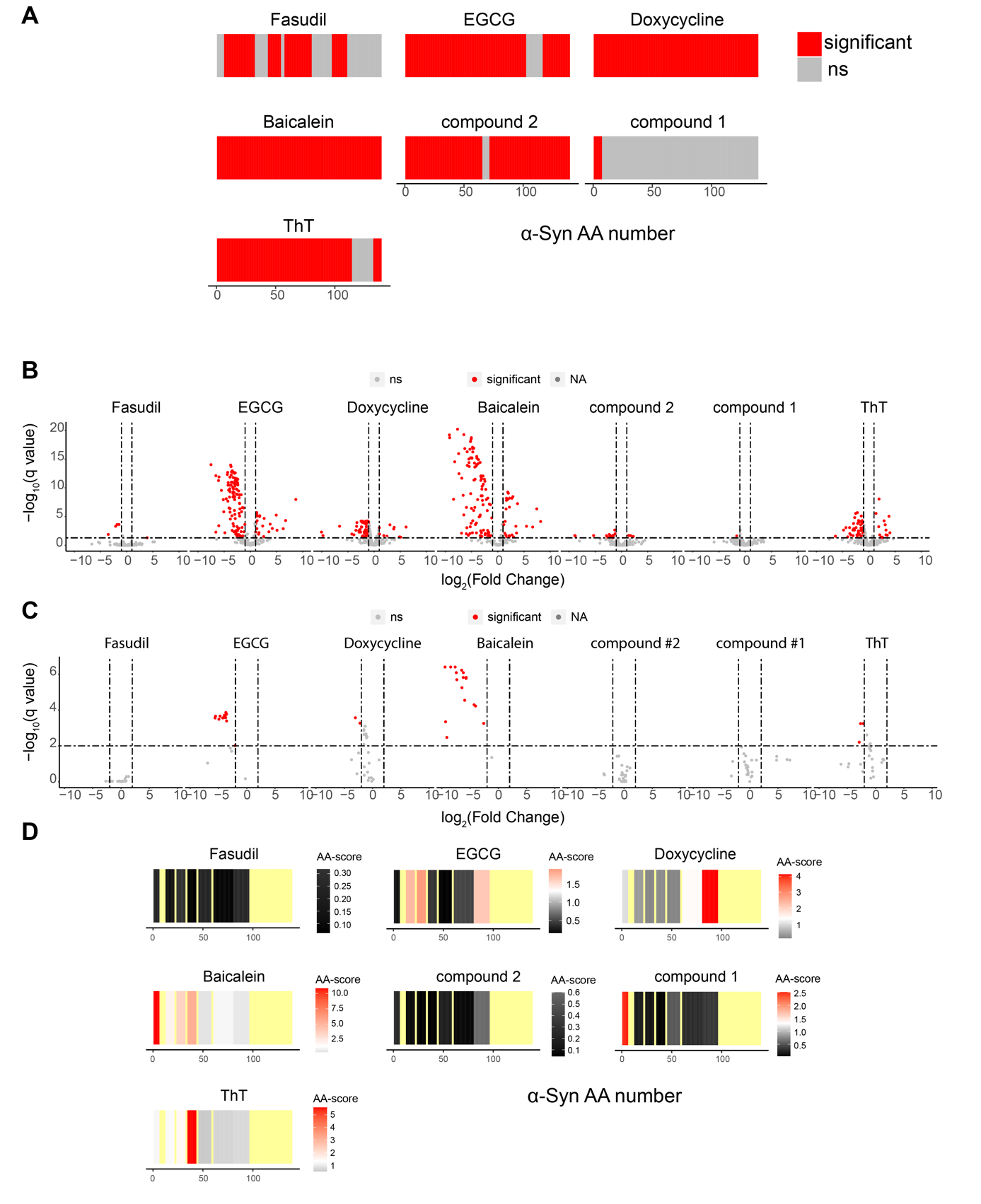


**Supplementary figure 9. Compound interactions with α-Synuclein fibril. A** Peptide analysis fingerprints of α-Synuclein treated with compounds (non-significant in light grey, significant in red, not detected in yellow). **B** Volcano plot of compound treated α-Synuclein fibril (non-significant in light grey, significant in red). **C** Volcano plot of the control intensities of compound treated α-Synuclein fibril (non-significant in light grey, significant in red). **D** Peptide fingerprint of the tryptic control normalised data (The scale indicates the score per peptide. The significance threshold of -log_10_(0.05) x log_2_(2) is shown in white, with red indicating higher scores. The more intense the red colour, the higher the score. Not significant in grey. Not detected in yellow).


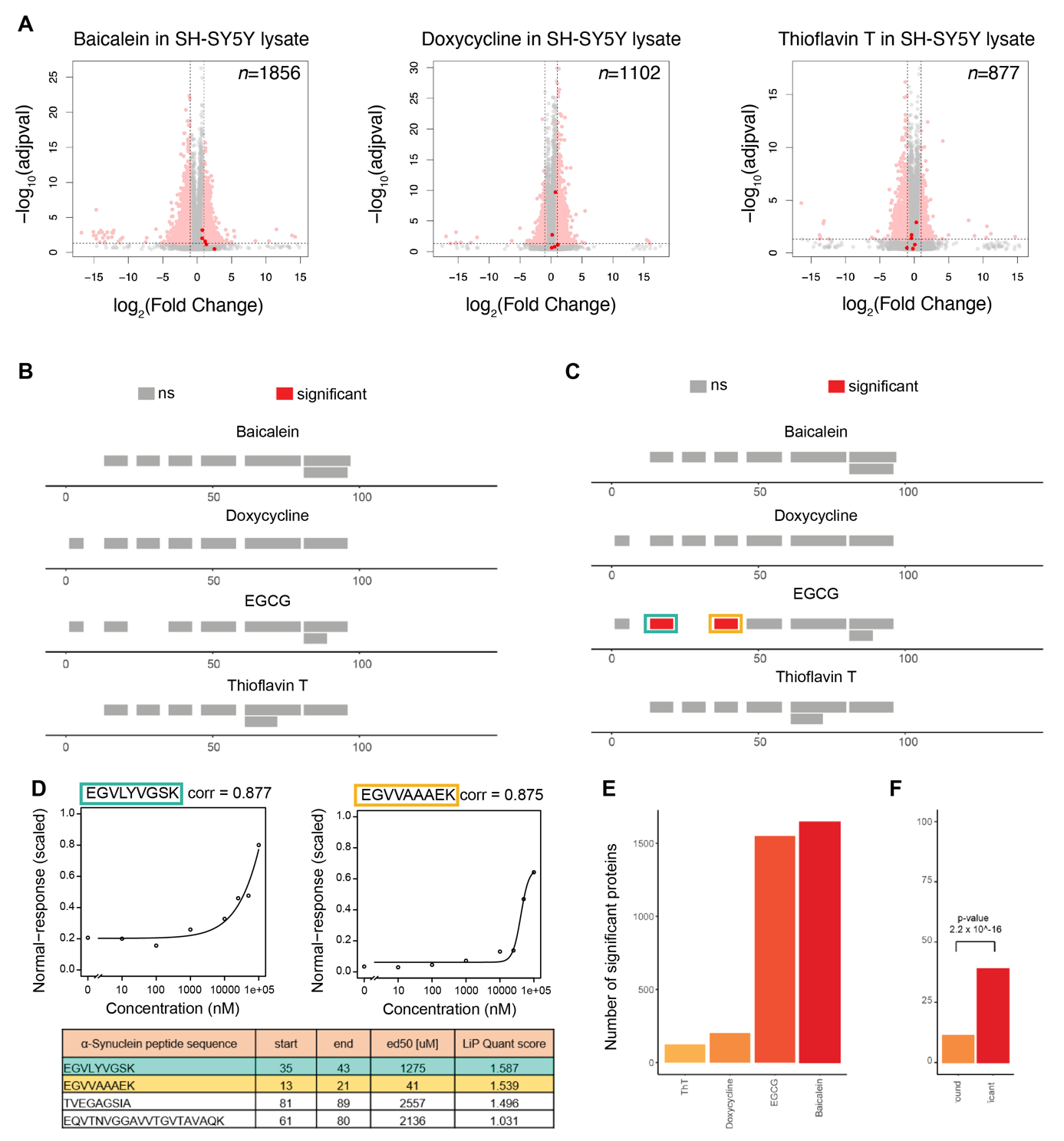


**Supplementary figure 10. A.** Volcano plots showing peptides with altered abundance after spike-in of the indicated compounds into cell lysates. Number of significant hits and total number of detected peptides are indicated. **B, C** The plots show a LiPQuant analysis of alpha-synuclein in the presence of indicated compounds, with detected peptides colored according to the LiPQuant score. Red is significant. Grey is not significant. Note that A and B are scaled differently; LiPQuant scores are shown to a threshold of 2.0 (B) and 1.5 (C). **D** The table shows α-Synuclein peptides and their start and end position, ec50 value and LiPQuant score. The blue and yellow colors indicate the two α-Synuclein peptides with LiPQuant score > 1.5 in the presence of EGCG . The plots show curve fits of peptide intensity versus EGCG concentration for the two α-Synuclein peptides with LiPQuant score > 1.5 in the presence of EGCG. **E.** Number of significant proteins in the presence of the indicated compounds, at LiPQuant score > 1.5. **F**. Fisher’s exact test of significant proteins (LiPQuant score > 1.5) detected in the presence of Doxycycline and in the Doxycycline pulldown experiment compared to not significant background.


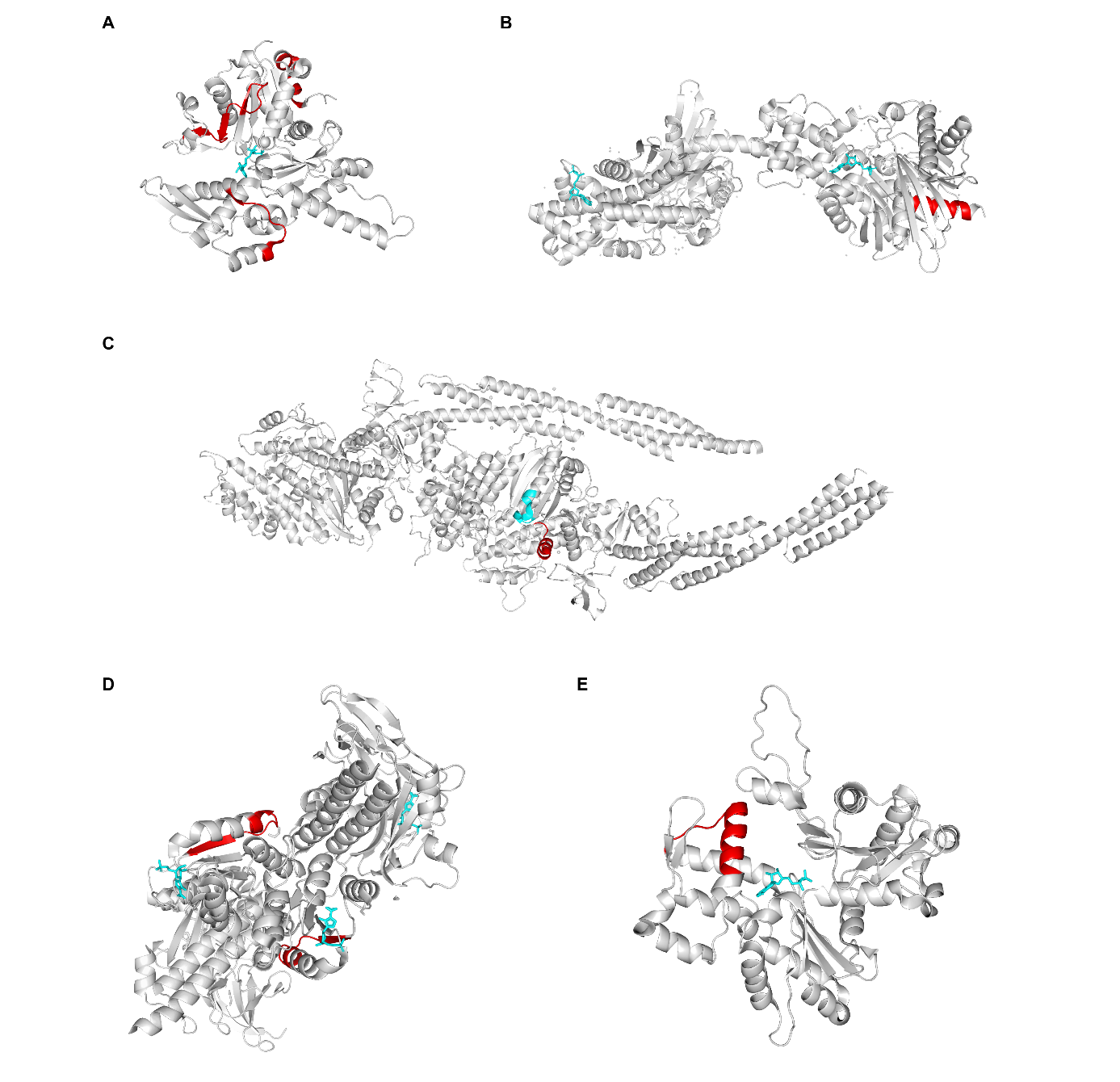


Supplementary figure 11. Significant hits with ATP/ADP/C2R bound pdb structures. A ACTR (pdb: 6uhc). Significant hits in red, ATP in cyan. B HK1 (pdb: 1dgk). Significant hit in red, ADP in blue. C MYH10 (pdb: 4pd3). Significant hit in red. ATP binding site from “uniprot.org” in cyan. D PAICS (pdb: 6yb8). Significant hit in red, C2R in cyan. E ACTB (pdb: 3j82). Significant hit in red, ADP in cyan.

**
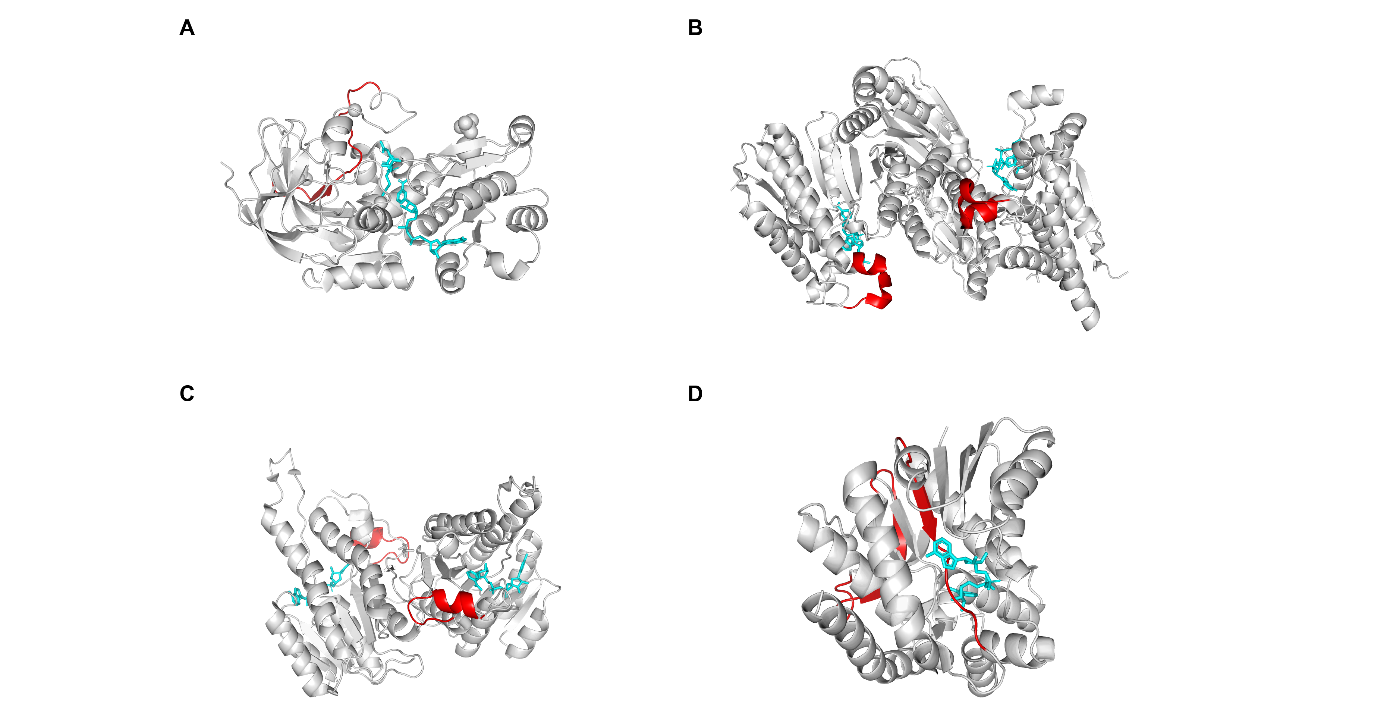
**

Supplementary figure 12. Significant hits with NAD/NDP bound pdb structures. A ADH5 (pdb: 1mc5). Significant hit in red, NAD and AHE in cyan. B IDH2 (pdb: 4ja8). Significant hit in red, NDP in cyan. C HSD17B10 (pdb: 2o23). Significant hit in red, NAD in cyan. D MDH2 (pdb: 2dfd). Significant hit in red, NAD in cyan.

*
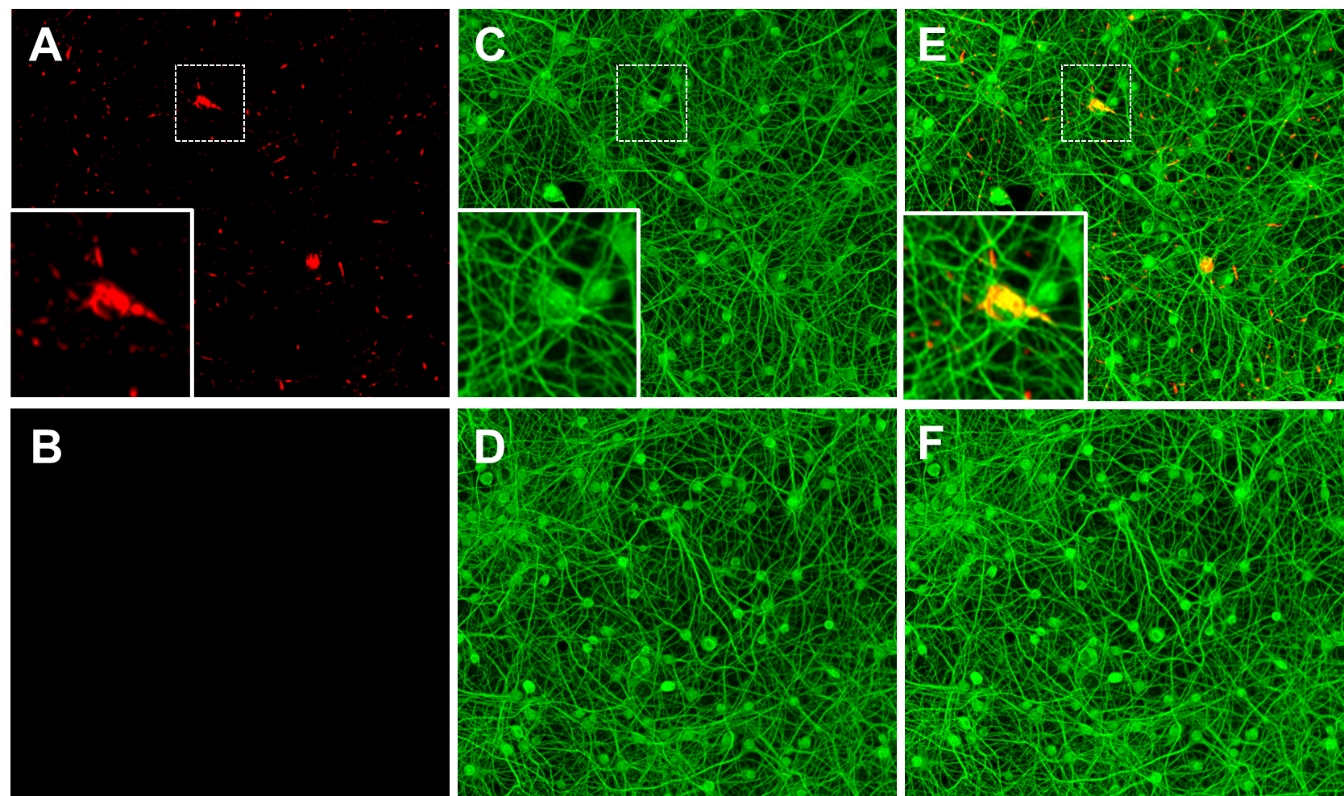
*

**Supplementary figure 13.** Fluorescence microscopy images of primary rat neurons treated with α-Synuclein PFFs. (A, B) pS129 was visualized in PFF-treated (A) and untreated (B) neurons by staining with anti pS129 α-Synuclein antibodies (1/300 dilution, ab51253). (C, D) MAP2 staining of PFF-treated (C) and untreated (D) neurons using anti-MAP2 antibodies (1/2200 dilution, ab5392). (E, F) merged view of pS129 and MAP2 channels of PFF treated (E) and untreated (F) neurons; inserts show higher magnification of area in dashed-line boxes.


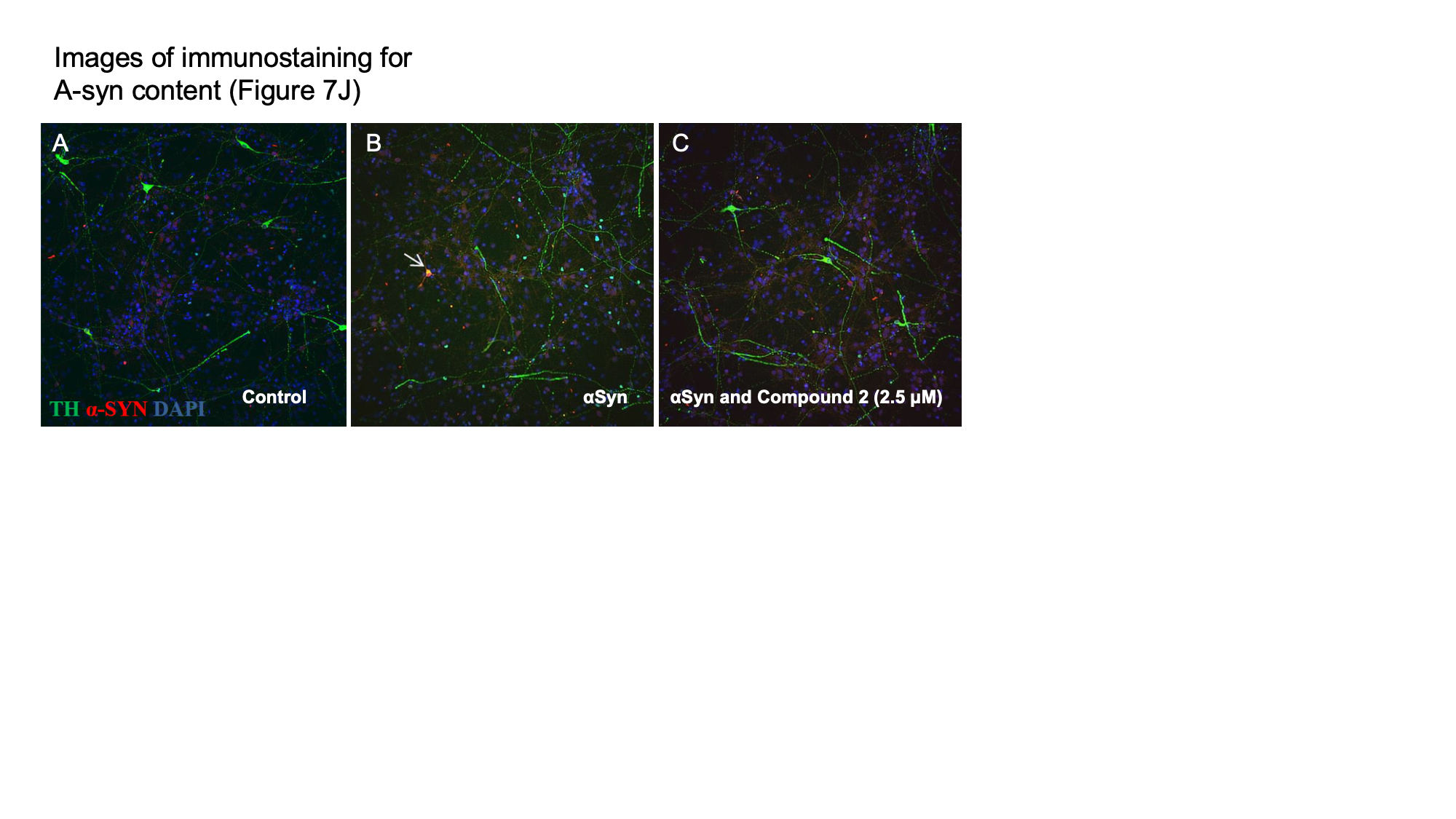


**Supplementary figure 14**. Fluorescence microscopy images of primary rat neurons stained with antibodies for tyrosine hydroxylase (TH) in green and α-Synuclein in red; nuclei are visualized by DAPI in blue. (A) Control, untreated neurons. (B) Neurons exposed to α-Synuclein PFFs. The arrow indicates a cell with co-localized α-Synuclein and TH. (C) Neurons exposed to α-Synuclein PFFs and treated with 2.5μM Compound 2.


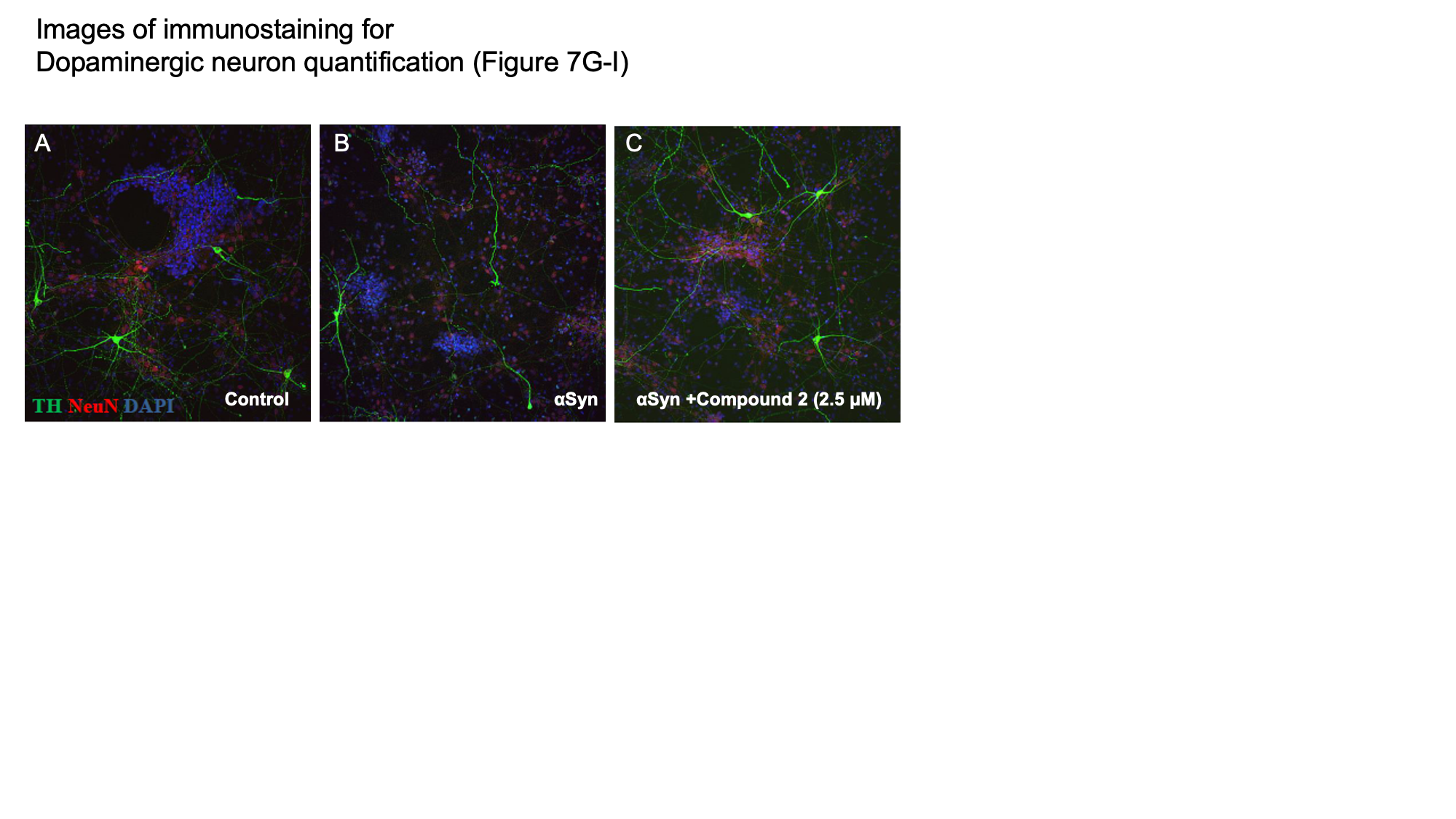


**Supplementary figure 15.** Fluorescence microscopy images of primary rat neurons stained with antibodies for tyrosine hydroxylase (TH) in green and NeuN in red; nuclei are visualized by DAPI in blue. (A) Control, untreated neurons. (B) Neurons exposed to α-Synuclein PFFs. (C) Neurons exposed to α-Synuclein PFFs and treated with 2.5μM Compound 2.
